# Experience of stress in parents of children with ADHD: A qualitative study

**DOI:** 10.1080/17482631.2019.1690091

**Published:** 2019-11-12

**Authors:** Sophie Leitch, Emma Sciberras, Brittany Post, Bibi Gerner, Nicole Rinehart, Jan M. Nicholson, Subhadra Evans

**Affiliations:** a School of Psychology, Deakin University, Burwood, Australia; b Murdoch Children’s Research Institute, Royal Children’s Hospital, Parkville, Australia; c Judith Lumley Centre, La Trobe University, Bundoora, Australia

**Keywords:** Attention deficit hyperactivity disorder, ADHD, parenting, stress, qualitative

## Abstract

**Purpose**: Qualitative research aimed at understanding the stress of parents of children with ADHD is limited and few interventions have been designed to directly target their stress. The study aim was to explore the stress of parents of children with ADHD using qualitative methodology.

**Methods**: Thirteen parents of children with ADHD participated in two focus groups. Open-ended questions explored parents’ experiences of stress. Focus groups were recorded, transcribed, and coded using thematic analysis. Parents also completed the Parenting Stress Index–Short Form.

**Results**: Four primary themes were identified: The child’s behaviour feels like a “wrecking ball”; Coping with the “war at home”; A divided family: “relationships don’t survive”; and Craving support: “it’s goddamn hard work”. Five of eleven participants who completed the PSI-SF scored in the clinically significant range indicating levels of stress that require professional support.

**Conclusions**: Parents attribute their high stress to their children’s behaviour, unmet needs for support, and social stigma. Parents request support to enable them to cope and appear to represent a clinical population who require mental health care and support themselves. Future interventions directly targeting the stress of parents of children with ADHD may provide wide-ranging benefits for their children and families.

## Introduction

Attention deficit/hyperactivity disorder (ADHD) is a common mental health disorder of children and adolescents, impacting an estimated 63 million children and adolescents worldwide (Polanczyk, Salum, Sugaya, Caye, & Rohde, 2015). With core symptoms of inattention and/or hyperactivity-impulsivity, ADHD is a chronic condition that interferes with an individual’s functioning (American Psychiatric Association AP, 2013). Associated health and behavioural issues are also common, including poor overall health (Sciberras et al., 2016), conduct problems, anxiety and depression (Angold, Costello, & Erkanli, 1999; Biederman et al., 2006). When experienced in childhood, ADHD adversely impacts the family system (Johnston & Mash, 2001). In addition to experiencing significantly more parenting stress than parents of typically developing children (Theule, Wiener, Tannock, & Jenkins, 2013), parents of children with ADHD are reported to experience greater levels of parenting stress than parents of children with autism (Miranda, Tárraga, Fernández, Colomer, & Pastor, 2015) and physical conditions such as HIV infection or asthma (Gupta, 2007). A large body of quantitative research has documented the severity of stress experienced by parents of children with ADHD, and emphasized the need to target this stress to improve both child and parent outcomes (Theule et al., 2013). However, qualitative research aimed at understanding the stress of parents of children with ADHD is limited and few interventions have been designed to directly target their stress (Treacy, Tripp, & Baird, 2005). As such, the present study employs a qualitative methodology to explore the stress of parents of children with ADHD to inform intervention development.

Parenting stress can be described as stress that arises when a parent’s perceptions of the demands of parenting exceed their resources to deal with them (Deater‐Deckard, 1998). Increased parenting stress is associated with numerous negative outcomes for children with ADHD and their parents, including: the worsening of a child’s ADHD symptoms, reduced response to intervention, reduced quality of the parent-child relationship and decreased parental psychological well-being (Johnston & Mash, 2001; Modesto-Lowe, Danforth, & Brooks, 2008; Theule et al., 2013). Parenting stress may impact children via a number of pathways, including poor monitoring of children’s activities and whereabouts, and increased use of corporal punishment and controlling rather than supportive parenting strategies (Deater‐Deckard, 2004; Rogers, Wiener, Marton, & Tannock, 2009a, 2009b; Wirth et al., 2017). Challenging child ADHD behaviours have also been suggested to interfere with the development of attachment security among children with ADHD (Clarke, Ungerer, Chahoud, Johnson, & Stiefel, 2002).

Extensive quantitative studies have documented the need to target the stress of parents of children with ADHD in order to improve both child and parent outcomes (Theule et al., 2013), although qualitative research exploring the stress experienced by parents of children with ADHD is limited. More broadly, there has been a growth of qualitative studies that consider parents of children with ADHD (Ahmed, Borst, Yong, & Aslani, 2014; Davis, Claudius, Palinkas, Wong, & Leslie, 2012; Firmin & Phillips, 2009; Frigerio & Montali, 2016; Ghosh, Fisher, Preen, & Holman, 2016; Laugesen & Groenkjaer, 2015; McIntyre & Hennessy, 2012; Moen, Hall-Lord, & Hedelin, 2014; Mofokeng & van der Wath, 2017; Moen, Hall-Lord, & Hedelin, 2011). Several studies have examined the parental role and family experience of living with a child with ADHD (Firmin & Phillips, 2009; McIntyre & Hennessy, 2012; Mofokeng & van der Wath, 2017). Studies have also explored the information provided to parents about ADHD, as well as parents’ decision-making about treatment options for their children (Ahmed et al., 2014; Brinkman et al., 2009; Davis et al., 2012; Ghosh et al., 2016). These studies have drawn attention to the difficulties that parents of children with ADHD experience, such as challenging child behaviours, difficulties attending to siblings when caring for a child with ADHD, and lack of support from professionals (Brinkman et al., 2009; Davis et al., 2012; Mofokeng, 2017; Moen et al., 2011). However, there has been limited primary focus on understanding parents’ wider sources and manifestations of stress in a desire to identify and support parents’ mental health needs. Consistent with this previous research, children are likely to play a role in this stress; however, parents may have additional sources of stress, including a high likelihood of having their own undiagnosed mental health issues, such as ADHD (Johnston & Mash, 2001). A broad understanding of parents’ stress and distress is valuable for informing intervention development, as parents may need help in broadly coping with life stress, or interventions may be better placed by targeting coping in relation to the child’s behaviours. As Australian voices have been underrepresented in the handful of existing studies that have captured the parenting stress of parents of children with ADHD, it is also important to add to the diversity of voices to understand whether there are cross-cultural differences in the stress experienced by parents. Additionally, as external validity in qualitative research can be developed by connecting the comparable findings of multiple independent researchers (Firmin & Phillips, 2009), extending the limited number of qualitative studies that capture the stress of parents of children with ADHD is of importance.

In this study, we aimed to understand the experiences of stress in Australian parents of children with ADHD who self-identified as being stressed. Specifically, we wished to understand parents’ views on the sources and manifestations of their stress, with the goal of identifying parents’ needs to inform future interventions designed to support parents and ultimately their children.

## Materials and methods

This qualitative study was undertaken to understand parents’ lived experiences of stress, namely the identification of stressors, and how they communicated their parenting stress experiences in their own words. Focus group interviews were used to take advantage of the expected dialogue between group members which, in turn, would lead to the discussion of a broader range of topics and experiences and provide a rich understanding of these parents’ experiences as a whole (Grudens-Schuck, Allen, & Larson, 2004).

### Participants

Participants were recruited from a database of parents of children formally diagnosed with ADHD who had agreed to be contacted about research studies. Forty-nine families with children aged 5–12 years were reached via phone. Of these, 25 families consented to receiving more information and met the following inclusion criteria of: having a child aged 5–12 years of age with a prior diagnosis of ADHD, parents experiencing stress as rated five or above using the Stress Numerical Rating Scale-11 (Karvounides et al., 2016) and speaking English. Twenty-four families did not meet inclusion criteria or did not wish to participate in the study. The primary reason parents did not meet inclusion criteria was due to their Stress Numerical Rating Scale-11 score being below five. Families raised the time commitment as the primary barrier to participation in the study. Of the 25 families who consented to receiving more information, 12 families were unable to participate in/attend the focus group interviews due to logistical reasons (including weekend work, their children’s sporting commitments and transport arrangements), although consented to be contacted for future ADHD research. Thirteen families indicated they were able to attend the focus group interview dates and provided written consent. Another wave of recruitment was planned with additional focus group interviews planned; however it was the view of the research team that there was no emergence of new knowledge after the second focus group interview. Themes from the first focus group interview (n = 8) were repeated in the second (n = 5). Therefore the research team agreed that inductive thematic saturation (Saunders et al., 2018) was reached after this initial wave of recruitment.

### Procedure

Ethical approval was granted by Deakin University’s ethics review board.

Two focus group interviews (with two meetings per group) were held at Deakin University over two weekends in May and June 2017 in Melbourne, Australia. Each participant was asked to attend four hours of focus groups interviews in total (two hours on a Saturday afternoon and two hours on a Sunday afternoon). Parents were compensated for their attendance with a $30 AUD gift voucher for attending the first day of the focus group interviews and a $60 AUD gift voucher for attending the second day. In an effort to give low income parents an opportunity to participate and to increase the diversity of voices, taxi fares to and from the sessions were paid for one low income family who indicated that they would otherwise be unable to attend.

Open-ended questions relating to stress were asked on the first day of each focus group interview and centred on: parents’ sources and feelings of stress (e.g., “What are the key sources of stress in your life?”), the effect of parents’ stress on their children (e.g., “What effect do you think it might have on your child?”), their children’s behaviours that contribute to stress (e.g., “What child behaviours ‘push your buttons’?”), and how parents manage their stress (e.g., “What have you done in the past to manage your stress?”). The researchers used follow-up questions to clarify responses and to elicit in-depth answers. Focus group interviews were audio recorded, with the researchers also making some supplementary field notes during the focus group interviews. The recordings were then transcribed verbatim with pseudonyms used in place of identifiable data, and then cross-checked by a separate transcriber for accuracy and uploaded to Nvivo 10 (2012) qualitative data analysis software (NVivo10, 2012). To supplement these qualitative data, participants also completed the Parenting Stress Index—Short Form (PSI- SF), a 36-item, validated and reliable measure of parenting stress (Abidin, 1995). Due to the nature of focus group interviews, it was not possible to match quotes to specific parents or to their scores on the PSI-SF.

The research team members were all female and included a combination of researchers, training psychologists and psychologists. In order to minimize researcher bias, one member of the research team who has specific expertise in ADHD *(ES)* was not present during the focus group interviews and did not have any direct contact with the participants. The focus group interviews were led by (*SE*), due to her expertise in qualitative research, with the support of co-facilitators *(SL), (BG)* and *(BP)*. Only the participants and the researchers were present during the focus group interviews. The participants were informed of the purpose of the research at the recruitment stage as well as during the first focus group interview. Each of the facilitators also introduced themselves (including their role and research interests) to the participants during the first focus group interview. Prior to the study commencement, the only contact the researchers had with the participants was for recruitment purposes.

### Data analysis

Data were analysed using thematic analysis, a systematic process of coding patterns and themes to examine a group’s understanding of a particular issue (Braun & Clarke, 2006). This involved finding units of meaning contained in single words, phrases or entire quotes in the text and tagging these units with codes which captured their meaning. Consistent with Braun & Clarke’s approach, the following data analysis steps were applied: familiarization with data; generation of initial codes; search for themes among codes; review of themes and defining and naming themes (Braun & Clarke, 2006). After four researchers (*SE, SL, BP, BG*) read the focus group interview transcripts and independently coded the transcripts, the four researchers discussed the codes to ensure consistency and accuracy of coding participants’ responses. Minor discrepancies identified in the language used by researchers to code the interviews were identified and resolved through discussion between the researchers before identifying themes.

The approach was inductive, such that the final themes captured an overall picture of stress in parents of children with ADHD, reporting their realities without filtering them through a theory (Braun & Clarke, 2006). The themes were validated based on whether their internal content cohered meaningfully and whether each theme captured distinct parts of the overall data set (Braun & Clarke, 2006). Given the inductive approach, an extensive literature review was not completed until after the themes were identified, in order to reduce researcher bias. The peer review process was used continuously to support the credibility of data analysis. A critical realist approach (Willig, 1999) was taken during the preparation and data analysis to ensure the analysis reflected what participants said about their lives, but that this was also placed within the Australian context.

## Results

### Participants

A total of 13 parents from 11 families attended two focus group interviews. Two families who had provided written consent did not attend.

All questionnaires were completed by mothers, therefore demographic information and PSI-SF data is not available for the two fathers who attended with their spouses. Parent and child characteristics are reported in Table I. Mothers’ ages ranged from 38 to 50 years, and the age of their diagnosed child was from 7 to 11 years. The mean age at which children were diagnosed was 6.4 years. Five of the eleven parents who completed the PSI-SF scored in the clinically significant range, indicating levels of stress that require professional support.

**Table I.** Parent and child characteristics.

	Parent Characteristics				Child Characteristics	
Group	Participant	Sex	Age	Education achieved	Sex	Age
1	1	F	40.8	University Degree	M	11.3
	2	F	38.6	University Degree	M	11.6
	3	F	40.6	University Degree	M	11.1
	4	M				
	5	F	38.8	Vocational Cert./Degree	F	10.9
	6	M				
	7	F	40.4	Vocational Cert./Degree	M	10.8
	8	F	45.2	University Degree	M	7.8
2	9	F	50.4	University Degree	F	11.8
	10	F	48.1	Vocational Cert./Degree	M	11.9
	11	F	41.1	Vocational Cert./Degree	M	7.9
	12	F	40.5	Year 12	M	9.0
	13	F	38.4	University Degree	M	7.5

M: Male, F: Female.

#### Qualitative findings

As displayed in Table II, data were categorized into four main themes reflecting experiences of the parents’ stress. Expressed parent quotes were included in the theme titles. The first theme: The child’s behaviour feels like a “wrecking ball” illustrates parents’ main message, that their child’s behaviour is the primary origin of their stress. Three subthemes were identified relating to key stress-provoking child behaviours and represent both the outer intensity and inner distressing aspects of child ADHD: “Uncontrollable outbursts”, “Child absentmindedness”, and “Child self-loathing”. The second theme: Coping with the “war at home” captures two subthemes: parents’ lived experience of parenting stress in a near constant state of hypervigilance, and parents’ resilience and coping through the stress. The third theme: A divided family: “relationships don’t survive” represents the family-level impact of their children’s behaviour, with two subthemes capturing the impact on siblings and partners. The fourth theme: Craving support: “it’s goddamn hard work” encapsulates two subthemes: parents’ unmet needs for support and how parenting a child with ADHD can be a form of social stress, where parents live with stigma and scrutiny.

**Table II.** Qualitative study findings.

Themes and subthemes
Theme 1: The child’s behaviour feels like a “wrecking ball”. Subtheme 1: “Uncontrollable outbursts”, Subtheme 2: “Child absentmindedness”, Subtheme 3: “Child self-loathing”
Theme 2: Coping with the “war at home” Subtheme 1: A state of hypervigilance Subtheme 2: Resilience through the stress
Theme 3: A divided family: “relationships don’t survive” Subtheme 1: Impact on siblings Subtheme 2: Impact on partners
Theme 4: Craving support: “it’s goddamn hard work” Subtheme 1: Social stigma Subtheme 2: Unmet needs

### The child’s behaviour feels like a “wrecking ball”

When discussing sources of stress in their lives, parents focused almost exclusively on their children’s ADHD behaviours as causing intense distress and difficulty. Three subthemes were presented, concerning distinct aspects of behaviour that were described as ripping through families, leaving parents exhausted.

#### Uncontrollable outbursts

Parents described the difficulties they experienced when dealing with their children’s outbursts which tended to be intense, extreme and frequent. Parents used highly expressive language when discussing these outbursts including: “ADHD rampage” and “when he has a meltdown, it’s like a volcano going off.” Parents shared stories of their children’s violence in the home, including punching walls and threatening siblings. Three parents mentioned chairs being thrown across the room and at them by their children. Parents perceived these outbursts as being intense and destructive. They also described experiencing a loss of control over their children’s behaviour.

Someone explained to us like you’ve let the handbrake off a car and it’s coming down the hill. You’re not going to stand in front of it. You’ve got to wait until it gets to the bottom of the hill and stops, then you deal with it. Like trying to step in front of them and stop a meltdown is a waste of your own energy.

#### Child absentmindedness

Parents also reported that their children’s absentmindedness was difficult to manage and was associated with feelings of doubt about their competence as parents. One parent stated:

To do eight or ten reminders to get dressed or do your teeth or something. After about the sixth or seventh you’re ready to just tear your hair out.

Highlighting the reciprocal nature of parent-child emotions and behaviours, another parent expressed how this behaviour was particularly difficult to deal with if the parent was stressed or in a hurry for example due to work:

[she] will leave her homework to the very, last, last minute, so that’s very difficult, or it will be that it’s just a bad night, I’ve had a bad day at work, it’s been a long day and there’s other things that need to get done, and she’ll be like “oh I need to go to Officeworks” and I go “it’s a quarter to nine! How long have you known? A week?”

#### Child self-loathing

Parents also shared their stress and sadness around their children’s expression of self-loathing as a result of having ADHD and associated social difficulties:

My son quite often likes to hit himself or [say] “I’m stupid, I’m silly”. Like that whole self-hatred I really struggle with. So it’s very hard to cope with that.

Two parents mentioned that their children wanted to die, with one mother sharing about her son: “Seven-years old and he’s like, oh I can’t do anything right I might as well kill myself. And which parent wants that?” and another sharing: “When you’ve got your kid on the street because they’re waiting for a car to hit them because their life sucks.”

Not all parents stated that their children verbalized such intense self-loathing, but many described their children’s sense of isolation resulting from a lack of social skills. One parent expressed feeling heartbroken that her son had withdrawn from his peers since they would become angry with him for being unable to keep up with the rules of their games. Another reported that in the past, her child had come home from school with black eyes and bruises from bullying due to his social difficulties.

### Coping with the “war at home”

Although parents attributed much of their stress to incidents involving their children’s behaviour, their stress remained high even when their children were not present or when their children were present and calm. It was as though parents were in a perpetual hypervigilant state with the anticipation of the potential behaviours of their children making them feel “on edge” and alert “24–7”, leading to a sense of emotional and physical exhaustion.

### A state of hypervigilance

One parent described her parenting experience as “being constantly mentally engaged” in order to remain one step ahead of her child. Parents expressed a state of self-monitoring in order to avoid triggering outbursts. Monitoring what they say, how they say it, and when they say it, parents went to great lengths to avoid upsetting their children. Many reported tiptoeing around their children, which was put as “tiptoeing but rushing” by one mother describing the urgency and sensitivity that was required of her. However, at least one parent took a more combative approach, describing the task of keeping one step ahead of their child as a fight: “You’re always fighting to show you’re tough, I’m the parent”.

Contributing to their hypervigilant state, the rules and strategies for parenting children with ADHD were ever-changing, which brought an additional sense of disequilibrium and urgency:

You’ll use a strategy yesterday and find that it worked really, really well. You’re confronted with the same situation today and that strategy is not working. So I have to think of something else quick.

The sense of monitoring and alertness extended to keeping the house in order, including completing household chores and errands to a timetable, so parents could readily respond to their children when he or she returned from school or when an outburst occurred:

So everything can be in order. So when something might happen, after school it could be, so then I’m not sort of pulling my hair out. So everything is done. At least I can engage with that new problem that just occurred. But I don’t have to think about the dinner or buying this or the shopping or whatever, everything’s done.

Other parents reported similar experiences, highlighting the demands placed on them to be organized and prepared, anticipating every aspect of the family’s needs with an almost military precision.

In addition to the stress of anticipating outbursts, many parents commented that it was difficult to contain their reactions during outbursts and reported spikes of extreme stress during incidents. Many parents resorted to “screaming and yelling”, “smacking” and “losing my shit” and expressed difficulty coping with the overwhelming emotions their children’s behaviour stirred in them, including intense anger:

So it’s that, that moment of time in between, where I just stop and go, okay I can go for a walk, go outside, I can go kill him, or I can go … sometimes you want to—kill … It’s not easy. I could slap (him) into the high heavens.

#### Resilience through the stress

A few parents reported developing coping strategies, which most commonly involved removing themselves from the situation. One mother described how she mowed the lawn in order to deal with her frustration at her child. Another mentioned a recent period of growth, where she had cultivated states of compassion and acceptance to deal with her child’s difficult behaviours:

I’ve come off that phase where I used to smack and I used to react … Somewhere along the line there’s been a little click in my brain where it’s like “stop worrying about everybody else, stop worrying about your feelings to some extent and YOUR expectations and standards and just think about that child that can’t control what they’re doing. And think about how they’re feeling. So I’ve taken away my own sort of selfish social standards as such and have tried to look at him in a different way. And then when I’ve allowed myself to do that and accept some things that I don’t like, the calmer I’ve become …

### A divided family: “relationships don’t survive”

Distressing child behaviours reverberated throughout the family. Parents discussed the way their children’s intense, unpredictable and stressful outbursts affected siblings and partners.

#### Impact on siblings

Impact on siblings was particularly worrisome to parents, who often had to help manage the reactions and stress of siblings:

I have a younger one, he gets quite upset by anything confrontational. So, I find that a big stress, trying to keep peace enough that he doesn’t get upset, too. Because he gets really sad if things get out of control. So that’s stressful for me, watching my little one get upset by an older kid, my oldest child’s outbursts. Yeah, that’s stressful.

Some parents expressed guilt that the sibling typically “gets forgotten about” and worry that siblings would grow up to feel neglected and become resentful due to the lack of attention. Additionally, parents reported that their stress negatively impacted all children in the family, including children with ADHD and siblings alike. One parent mentioned that her stress put the children “on edge and … they’re gonna start fighting, as they do, quite frequently”. Another stated that when she feels stressed, she loses her temper more easily with all family members.

#### Impact on partners

In addition to impacting siblings, the presence of ADHD within the family negatively impacted marital relationships. One couple discussed how the “nervous tension” in the house sometimes lead to a “spat” between them, and another described how completing the parenting stress questionnaire as part of this research prompted her and her husband to have “a massive discussion” on how their child’s ADHD behaviour had placed stress on their relationship, which she had not previously realized due to her focus on managing their child’s behaviour. Another commented:

And there’s a lot of single parent families too because the stress of having a child like that on your relationship is massive and unfortunately a lot of relationships don’t survive it.

### Craving support: “it’s goddamn hard work”

Parents felt that raising a child with ADHD was too difficult for any individual family to cope with:

Well it takes a village to raise a child, and I think with us too we need more support than most parents. You know because it’s goddamn hard work.

Despite the need for such support, parents expressed that they frequently dealt with the dual blow of criticism from others, and a lack of resources, which represents the two sub-themes identified: social stigma and unmet needs.

#### Social stigma

Parents reported substantial distress from the difficulty of living with family and societal judgements related to their child’s diagnosis, behaviours and medication use. Parents expressed that they felt “isolated” and “ostracized” because the public did not understand ADHD, its consequences, and the value of medication. Parents shared that they had encountered highly vocal individuals who were harsh in their criticisms, commenting that ADHD was a joke, the parent was overmedicating their child, and making judgements about the parent’s behaviour:

I’ve been told that I’m a lazy parent … I’m lazy because my kids are highly medicated. If I learned to control my kids there wouldn’t be a problem.

Another parent described the “horrible” experience of a fellow parent telling her to put her son, who has an IQ of 120 but suffers from social and communication difficulties, into a special needs school. Parents explained how a poor understanding of ADHD extended into some of their familial relationships. One mother commented that a family member recommended that she “just keep belting them, you’ll belt it out of them”. A number of parents also described how their cultural backgrounds impacted the stigma they experienced from their families.

#### Unmet needs

Parents described an unmet need for support and coping “apart from coffee and Valium”. The need for support included a more educated and understanding public which extended to the medical and education settings. One parent identified the challenges of providing even routine medical care for her son, who needs to be sedated for blood tests. Yet, medical staff frequently discredit her parental experience, and a recent routine blood draw turned into an all-day ordeal with her son requiring general anaesthetic and being held down by eight hospital staff in order to complete the test, because she was not believed that he would need sedation. Another parent described unmet needs for understanding from school staff, who could be judgemental and lacked empathy towards her child and his communication difficulties:

When he gets angry he’ll start hissing like a snake or making grunting noises … The teachers say, “Is he being raised by dogs or something?”. And I’m like “No, he’s just telling you ‘get out of his space, leave him alone’” … The school doesn’t seem to be helping him … .

Another parent agreed that children with ADHD were not well catered for in schools because ADHD was not seen as a “serious disorder”. She stated that children were “falling between the cracks … they’re not bad enough and they’re not good enough to get by on their own”.

Parents also noted a lack of resources for parents like them, and a lack of recognition that they are a group in need. Parents identified the availability of resources for common co-morbidities, such as oppositional defiance disorder and autism, but support groups and skills training were largely absent for parents of children with ADHD. All expressed a keen interest in participating in any programme that would help their families and their relationships with their children.

Parents’ expressed distress related to isolation and stigma, and frustration regarding the lack of supports. Their sense of relief in sharing experiences and discussing topics with other parents who understood them was observed across the groups. Parents described feeling connected and that hearing others’ experiences helped them feel validated in their own experiences and challenges. After the focus group interviews, parents provided each other with contact details, intending to stay in touch.

## Discussion

This study aimed to understand the stress experienced by parents of children with ADHD. Parents attributed their high levels of stress to their children’s behaviour and social difficulties, their unmet needs for support, and their experiences of social stigma. By providing detailed insight into the stress of Australian parents of children with ADHD, this study enriches the large body of quantitative literature indicating that parents of children with ADHD display high levels of parenting stress (Theule et al., 2013). This study also builds upon the growth of qualitative studies that more widely consider parents of children with ADHD (Ahmed et al., 2014; Davis et al., 2012; Firmin & Phillips, 2009; Frigerio & Montali, 2016; Ghosh et al., 2016; Laugesen & Groenkjaer, 2015; McIntyre & Hennessy, 2012; Moen et al., 2014; Mofokeng & van der Wath, 2017; Moen et al., 2011).

Qualitative studies provide a rich exploration of the experiences and perspectives of individuals (Braun & Clarke, 2014). The qualitative design of the present study is valuable in developing a deeper understanding of the parenting stress of parents of children with ADHD, as scores on measures may not adequately capture parents’ lived experiences. For example, despite the wide use of the PSI-SF as a measure of parenting stress, a recent study has suggested that the 85^th^ percentile cut off score (suggested to indicate clinically significant levels of parenting stress that require professional support) led to the exclusion of mothers experiencing difficulties who were in need of services (Barroso, Hungerford, Garcia, Graziano, & Bagner, 2016). Consequently, it has been recommended that clinicians consider lower cut off scores and additional factors when considering families’ requirements for professional support (Barroso et al., 2016). This was illustrated in the present study with six of the eleven parents who completed the PSI-SF scoring in the normal range, and five scoring in the clinically significant range indicating a need for professional support. These scores however were unable to capture the richness of the qualitative data collected from parents, who each described their experience of hardship and their stressors as a parent of a child with ADHD.

As only a handful of qualitative studies have captured the parenting stress of parents of children with ADHD, this study adds to the diversity of voices captured within the few international studies that exist. Rather than using theory or prior research to guide our analysis, an inductive approach was employed in this study to allow parents’ experiences to speak for themselves. Although our analysis was not biased by themes in prior research, the findings of our Australian study were generally congruent with the experiences of parents from the US, Norway and South Africa (Brinkman et al., 2009; Davis et al., 2012; Mofokeng, 2017; Moen et al., 2011). This implies a “shared experience” of the parenting stress of parents of children with ADHD which occurs despite the cultural differences and varied healthcare systems of these countries.

A strength of the present study was the focus on parents’ broader experience of stress, rather than focusing more narrowly on the impact of their child’s ADHD behaviour. Nonetheless, when asked open-ended questions about the stress in their lives, most Australian parents in this study discussed exclusively their children’s ADHD behaviour, rather than work or other life stressors, indicating that their children’s behaviour and their role as a parent was the primary source of stress. Such findings are consistent with previous qualitative studies that have captured the persistent daily caregiving burden parents of children with ADHD experience due to their children’s ADHD behaviours (Brinkman et al., 2009; Mofokeng & van der Wath, 2017). Australian parents’ descriptions of overwhelming stress and difficulty coping with their children’s outbursts were shared by parents in previous qualitative studies who highlighted their difficulty in coping with unpredictable behaviour such as temper tantrums, and challenges with everyday tasks such as following instructions and getting ready to leave the house (Mofokeng & van der Wath, 2017; Moen et al., 2011). In an attempt to cope, Australian parents in this study reported resorting to corporal punishment techniques that have previously been described by South African and US parents (Brinkman et al., 2009; Mofokeng & van der Wath, 2017).

While previous studies have noted the externalizing behaviour of children with ADHD to be a key source of parenting stress (Mofokeng & van der Wath, 2017; Moen et al., 2011), the findings of the present study also highlight the distress parents experience due to their children’s comorbid internalizing behaviour, including desire to self-harm. The distress parents experience due to their children’s comorbid internalizing behaviour is largely unreported in previous qualitative studies. However, quantitative studies have identified an increased prevalence of comorbid internalizing symptoms among children with ADHD (LeBlanc & Morin, 2004), which significantly increases parenting stress (Theule et al., 2013). As parents of children with comorbid conditions were not excluded from this study, this finding is unsurprising. Australian parents’ experiences of stress were likely influenced by their children’s comorbidities. However, as 64% of children with ADHD have at least one comorbid mental health condition (Jensen et al., 2001), this is considered a true reflection of parenting a child with ADHD.

As described by parents in the present study, the impact of children’s ADHD is felt across the family, emphasizing the need for healthcare systems and professionals to consider the whole family, rather than solely focusing treatment and services on the individual with ADHD. This need to address challenges within the family and broader systems of children diagnosed with ADHD has been discussed in the recent literature (Timimi, 2017). Parents in the US and Norway have described similar difficulties experienced by family members. For example, siblings often have to act as caregivers who are required to grow up quickly to cope with their sibling’s ADHD (Brinkman et al., 2009; Davis et al., 2012; Mofokeng & van der Wath, 2017; Moen et al., 2011). Like Australian parents, Norwegian and South African parents have recalled the significant challenges of attending to other children and family responsibilities, whilst trying to cope with the burden of parenting a child with ADHD (Mofokeng & van der Wath, 2017; Moen et al., 2011). Similar to our findings, previous studies have also noted marital conflict and disagreement due to couples’ differences of opinions about their children’s ADHD diagnosis and treatment (Brinkman et al., 2009; Mofokeng & van der Wath, 2017). Although no participants in this study reported questioning the ADHD diagnosis of their child, the majority reported relationship conflict regarding their children’s ADHD diagnosis and treatment. Thus, future studies could explore differences of opinions about ADHD diagnosis and treatment within couples, and how this influences parent stress and coping.

In practice, considering the needs of the whole family may begin with screening the parents and siblings of children with ADHD for their own mental health issues. Such an approach is consistent with the practice guidelines from the Canadian ADHD Resource Alliance (CADDRA), and the UK’s National Institute for Health and Clinical Excellence (NICE), which are recommended by The Royal Australian and New Zealand College of Psychiatrists (RANZCP) (The Royal Australian and New Zealand College of Psychiatrists: Adult attention deficit hyperactivity disorder (ADHD) practice guidelines). These guidelines recommend screening and referring parents of children with ADHD for treatment of their own mental health conditions (Canadian ADHD Resource Alliance, 2018; NICE guideline [NG87] Attention deficit hyperactivity disorder: diagnosis and management), and recognize that targeting parent mental health can lead to improved parenting and supports children with ADHD (Canadian ADHD Resource Alliance, 2018). Australia’s National Health and Medical Research Council (NHMRC) guidelines released in 2012 also recommend support for families of children with ADHD including counselling, parenting education and respite care (National Health and Medical Research Council, 2012). However, despite these guidelines, parents in this study explicitly expressed their families’ unmet needs within the existing Australian healthcare system. Parents felt that limited support was available to children or parents given that in Australia ADHD is not considered a developmental disability to attract the same level of supportive services and funding as other childhood conditions such as autism. These findings have implications for healthcare systems that manage children with ADHD, such as requiring the necessary resources to adequately support and treat the families of children with ADHD. Additionally, these findings have implications for professionals and clinicians who work directly with families of children with ADHD, such as tailoring care to meet the specific needs articulated by families.

Australian parents’ distress due to community judgements and misunderstanding about their children’s behaviour, ADHD diagnosis, medication use, as well as their own parenting practices, appeared consistent with prior studies (Brinkman et al., 2009; Mofokeng & van der Wath, 2017; Moen et al., 2011). Norwegian parents have described health professionals and schools adding to their stress rather than helping to alleviate it, and feeling that professionals question their parenting practices rather than meeting or addressing their needs (Moen et al., 2011). Parents have also reported feelings of isolation from their friends and families, due to other adults being intolerant of their children’s behaviour (Mofokeng & van der Wath, 2017; Moen et al., 2011). Like US parents (Brinkman et al., 2009), Australian parents recalled the painful experience of friends and family members suggesting corporal punishment as a solution to their children’s behaviour.

The results of this study and similar qualitative studies indicate that parents of children with ADHD find parenting to be a stressful and fraught experience. Parents of children with ADHD have described loving, but not liking their children, and feelings of despair, sorrow, guilt, grief, anger, helplessness, depression, frustration, isolation and judgement are common (Brinkman et al., 2009; Davis et al., 2012; Mofokeng & van der Wath, 2017; Moen et al., 2011). The difficulties inherent in raising children with ADHD are likely to negatively impact parents’ psychological health, as well as their parenting practices and children (Theule et al., 2013). High levels of parenting stress are associated with both an increased risk of parental psychopathology (Kazdin, 1995) and maladaptive parenting practices, both of which are known to influence the course of children’s ADHD (Johnston & Mash, 2001; Modesto-Lowe et al., 2008). Interventions targeting parenting stress are therefore likely to address the parent’s mental health needs, as well as children’s needs (Theule et al., 2013).

Unlike other developmental disorders such as autism for which there are numerous programmes to reduce parent stress (Keen, Couzens, Muspratt, & Rodger, 2010; Samadi, McConkey, & Kelly, 2013; Tonge et al., 2006), few interventions have been designed to directly target the stress of parents of children with ADHD (Treacy et al., 2005). Descriptions of “constant” stress, with “heightened periods of extreme stress”, convey a need to provide parents with tools supporting a general baseline sense of calm, in addition to support during critical moments of extreme behaviours. Therefore, it is encouraging that some existing behavioural parent training programmes have identified reductions in parenting stress (Gerdes, Haack, & Schneider, 2012; Heath, Curtis, Fan, & McPherson, 2015). Research has also begun into the development and assessment of targeted stress management programmes for parents of children with ADHD (Parand et al., 2010; Sharif, Zarei, Shooshtari, & Vossoughi, 2015; Treacy et al., 2005); however, further research is required to establish the long-term efficacy and acceptability of these interventions for parents. In addition, further research is needed to understand the type of interventions parents are most receptive to, and the barriers and enablers to implementing interventions that specifically target the parenting stress of parents of children with ADHD within Australia’s healthcare system. Future research with parents of children with ADHD will ideally explore with parents how we can best support them and subsequently, their children’s functioning.

### Limitations

There are several limitations to this study. Firstly, due the recruitment of parents who self-identified as experiencing stress, this study is likely biased towards higher stress families. This recruitment decision was due to our aim of exploring and understanding the experiences of stress in Australian parents of children with ADHD, with the goal of informing future interventions to reduce parenting stress. Secondly, as noted in the methods section, it was not possible to match quotes to specific parents, and to their scores on the PSI-SF. Comparing individual parents’ descriptions of their stress to their scores on the PSI-SF would have been valuable.

Despite efforts to recruit fathers of children with ADHD, including requesting mothers to encourage their children’s father to participate, there remained challenges recruiting fathers. Although the two fathers’ voices have been utilized in the findings of this paper, our themes may be more reflective of mothers’ than fathers’ experiences. Similar challenges involving fathers of children with ADHD in research and clinical settings have been discussed previously (Singh, 2003). As mothers and fathers of children with ADHD may view parenting a child with ADHD differently (Singh, 2003), future studies should elucidate whether mothers and fathers of children with ADHD experiences stress differently. Similarly, as the majority of parents in this study had sons with ADHD, the findings are likely more reflective of parenting boys with ADHD. Given prior quantitative research suggests an association between parent stress and child gender (Theule et al., 2013), future qualitative studies should explore whether child gender impacts parents’ experience of stress.

As participants had children aged between 7–11 years, specific issues may arise when parenting teenagers or younger children with ADHD that were not captured here. Additionally, racial or ethnic background was not collected in the demographic questionnaire, which may have provided helpful context for the interpretation of study results. However, participants did disclose a number of different ethnic backgrounds in the focus group interviews including Greek, Maori and Croatian. Similarly, the collection of more detailed information about the parents (e.g., relationship status, mental health history) and their children with ADHD (e.g., symptoms, comorbidities etc.) would have been helpful for the interpretation of study results. Additional information about comorbid conditions would have been particularly valuable as some of the behaviours described by parents may reflect comorbid conditions (e.g., conduct disorder, depression). However, given 64% of children with ADHD have at least one comorbid mental health condition (Jensen et al., 2001), parents’ experiences of comorbid conditions are likely an accurate reflection of parenting the majority of children with ADHD.

### Conclusions

Australian parents of children with ADHD described their lived experience as a “war”, with a constant state of background stress interjected by major spikes in extreme stress. Parents identified their children’s behaviour and their parenting role as the primary source of their stress, with intense feelings of stigma and scrutiny perceived from professionals and their community. Parents expressed the family level impact of their children’s ADHD, and their unmet needs for support from Australia’s healthcare system and their community. Parents called out for support to enable them to cope with the “warzone” they experience and appear to represent a clinical population who are in need of mental health care and support themselves. Future interventions that specifically target the parenting stress of parents of children with ADHD and are designed with parents to meet their articulated needs, would likely provide wide-ranging benefits to individual parents as well as flow on effects to children.

## Data Availability

The data that support the findings of this study are available on request from the corresponding author. The data are not publicly available due to them containing information that could compromise research participant privacy.
